# Supplementary material for: Causal Relationship between Aging and Anorexia Nervosa: A White-Matter-Microstructure-Mediated Mendelian Randomization Analysis
Source: Biomedicines. 2024 Aug 16;12(8):1874. doi: 10.3390/biomedicines12081874 (PMC11351342; doi:10.3390/biomedicines12081874)
Supplement: Supplementary file 1 [file biomedicines-12-01874-s001.zip › Supplementary Figure/Figure S7/Single SNP plot of MR analysis for ubm-b-1946 to AN.pdf]

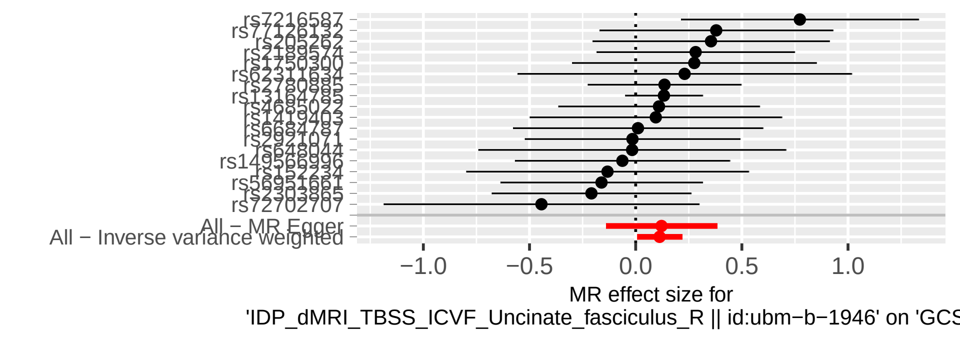

rs7216587
rs77126132
rs205262
rs2189574
rs1750300
rs62311634
rs2780885
rs13164785
rs4685022
rs1419403
rs6684787
rs2921071
rs648044
rs149566996
rs152234
rs56951661
rs2303865
rs72702707
All − MR Egger
All − Inverse variance weighted
−1.0
−0.5
0.0
0.5
1.0
MR effect size for
'IDP_dMRI_TBSS_ICVF_Uncinate_fasciculus_R || id:ubm−b−1946' on 'GCS
